# MEK inhibition exerts temporal and myeloid cell-specific effects in the pathogenesis of neurofibromatosis type 1 arteriopathy

**DOI:** 10.1038/s41598-021-03750-6

**Published:** 2021-12-21

**Authors:** Rebekah Tritz, Farlyn Z. Hudson, Valerie Harris, Pushpankur Ghoshal, Bhupesh Singla, Huiping Lin, Gabor Csanyi, Brian K. Stansfield

**Affiliations:** 1 Vascular Biology Center, Augusta University, Augusta, GA USA; 2 Department of Pharmacology and Toxicology, Augusta University, Augusta, GA USA; 3 Division of Neonatology, Department of Pediatrics, Medical College of Georgia at Augusta University, Augusta University, 1120 15th St, BIW6033, Augusta, GA 30912 USA

**Keywords:** Carotid artery disease, Translational research

## Abstract

Mutations in the *NF1* tumor suppressor gene are linked to arteriopathy. *Nf1* heterozygosity (*Nf1*+/–) results in robust neointima formation, similar to humans, and myeloid-restricted *Nf1*+/– recapitulates this phenotype via MEK-ERK activation. Here we define the contribution of myeloid subpopulations to NF1 arteriopathy. Neutrophils from WT and *Nf1*+/– mice were functionally assessed in the presence of MEK and farnesylation inhibitors in vitro and neutrophil recruitment to lipopolysaccharide was assessed in WT and *Nf1*+/– mice. Littermate 12–15 week-old male wildtype and *Nf1*+/– mice were subjected to carotid artery ligation and provided either a neutrophil depleting antibody (1A8), liposomal clodronate to deplete monocytes/macrophages, or PD0325901 and neointima size was assessed 28 days after injury. Bone marrow transplant experiments assessed monocyte/macrophage mobilization during neointima formation. *Nf1*+/– neutrophils exhibit enhanced proliferation, migration, and adhesion via p21^Ras^ activation of MEK in vitro and in vivo. Neutrophil depletion suppresses circulating Ly6C^low^ monocytes and enhances neointima size, while monocyte/macrophage depletion and deletion of *CCR2* in bone marrow cells abolish neointima formation in *Nf1*+/– mice. Taken together, these findings suggest that neurofibromin-MEK-ERK activation in circulating neutrophils and monocytes during arterial remodeling is nuanced and points to important cross-talk between these populations in the pathogenesis of NF1 arteriopathy.

## Introduction

Neurofibromatosis type 1 (NF1) is a common dominantly inherited cancer predisposition syndrome affecting 1 in 3000 persons that results from heritable and de novo loss-of-function mutations in the *NF1* tumor suppressor gene^1^. Inactivating mutations in *NF1* result in decreased expression of neurofibromin, which functions as a GTPase for p21^ras^ (Ras) to negatively regulate canonical MEK-ERK signaling^1,2^. Due to the ubiquitous expression of neurofibromin, germline mutations in *NF1* result in manifestations affecting every organ system although the timing and severity of individual manifestations vary even among families harboring the same *NF1* mutation.

Cardiovascular disease is common in persons with NF1 and is an important contributor to the excess mortality observed in this population^3^. A hallmark of NF1 is the development of early and severe arterial stenosis and aneurysms distributed throughout the arterial network. Similar to humans with NF1, mice harboring a single germline *Nf1* mutation (*Nf1*+/–) develop larger neointimas than littermate controls using a variety of injury models^4–6^. Although neurofibromin is expressed in vascular wall cells, neither endothelial cell (EC) or vascular smooth muscle cell (VSMC)-restricted *Nf1* heterozygosity result in the same robust neointima observed in mice carrying a germline *Nf1* mutation^6^. However, transfer of *Nf1*+/– bone marrow into WT mice amplified neointima formation while transfer of WT bone marrow into *Nf1*+/– mice suppressed neointima formation suggesting that bone marrow-derived hematopoietic cells drive neointima formation in *Nf1*+/– mice^6^. In support of this line of thinking, myeloid-restricted (LysMcre) *Nf1* heterozygous mice develop robust neointimas that mimic observations in *Nf1*+/– mice and point to a critical role for neurofibromin in myeloid cell function and their contribution to vascular injury and arterial remodeling^5^. Further, administration of a specific MEK inhibitor prevented neointima formation in *Nf1*+/– mice via a dose-responsive relationship^7^. Strikingly, commencement of the MEK inhibitor treatment one week after arterial injury lead to an even more potent suppression of neointima size in *Nf1*+/– mice and significantly reduced the number of myeloid cells (e.g. neutrophils and macrophages) within *Nf1*+/– neointimas^7^. Taken together, these observations strongly suggest that perturbations in neurofibromin-MEK-ERK signaling promote myeloid-cell mediated VSMC proliferation and arterial stenosis, but little is known about the interaction between discrete myeloid subpopulations and VSMCs and whether targeting individual populations is advantageous to prevent or treat NF1 arteriopathy.

In this study, we use in vitro and in vivo techniques to examine the interplay between myeloid derivatives in the evolution of arterial stenosis and determine how MEK activation controls neutrophil and monocyte/macrophage function in the pathogenesis of NF1 arteriopathy. Our findings suggest that neutrophils exert protective effects against neointima formation by maintaining circulating Ly6C^low^ monocytes within the vascular compartment and that neutrophil depletion or early targeting of MEK activation in neurofibromin-deficient neutrophils after arterial injury enhance neointima formation. Further, we demonstrate that monocytes/macrophages are the major drivers of arterial stenosis in *Nf1*+/– mice.

## Results

### Neurofibromin controls neutrophil function via MEK activation in vitro and in vivo

**Figure 1:**
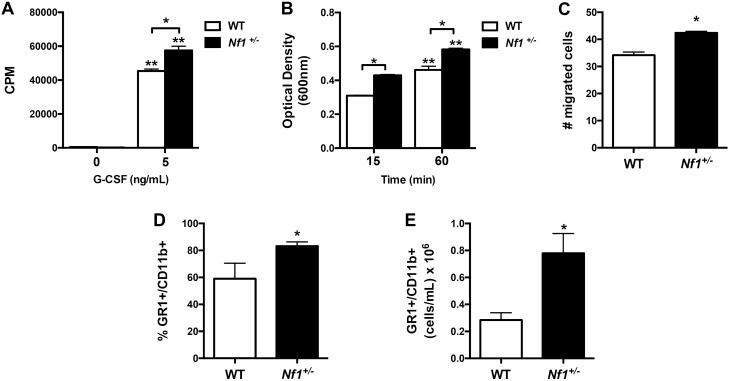
Neurofibromin controls neutrophil function in vitro and in vivo. (**A–C**) Quantification of WT (white bars) and *Nf1*+/– (black bars) neutrophil proliferation  (**A**), adhesion (**B**), and migration (**C**) in response to G-CSF. (**A**) Data represent thymidine incorporation reported as mean counts per minute (cpm) ± SEM, n = 4–5. *P < 0.01 for WT versus *Nf1*+/– neutrophils. **P < 0.001 for WT and *Nf1*+/– neutrophils without growth factor versus WT and *Nf1*+/– neutrophils incubated with G-CSF.  (**B**) Data represent mean optical density (600 nm) ± SEM, n = 4–5. *P < 0.001 for WT versus *Nf1*+/– neutrophils at each time point. **P < 0.001 for WT and *Nf1*+/– neutrophils at 15 min versus WT and *Nf1*+/– neutrophils at 60 min. (**C**) Data represent mean number of migrated cells ± SEM, n = 4–5. *P < 0.01 for WT versus *Nf1*+/– neutrophils. (**D **and** E**) Quantification of percent (**D**) and absolute number (**E**) of GR1 + /CD11b + neutrophils in peritoneal lavage fluid 48 h after LPS injection. (**D**) Data represent mean percent ± SEM, n = 4–5. *P < 0.01 for WT versus *Nf1*+/– mice. (**E**) Data represent mean number of cells per mL ± SEM, n = 4–5. *P < 0.001 for WT versus *Nf1*+/– mice. Analysis by 2-way ANOVA and Student’s *t* test.

We and others have demonstrated that neurofibromin-deficient monocytes and macrophages exhibit a distinct pro-survival phenotype^5,7–10^, however, the role of neurofibromin in other differentiated myeloid populations, including neutrophils, has not been described. Based on the observation that myeloid-restricted loss of *Nf1* results in robust neointima formation and the presence of neutrophils in early and mature *Nf1*+/– neointimas, we derived neutrophils from the bone marrow of WT and *Nf1*+/– mice and characterized their function. In response to granulocyte colony-stimulating factor (G-CSF), *Nf1*+/– neutrophils exhibit a marked increase in proliferation, adhesion, and migration when compared with WT neutrophils (Fig. 1A–C). Next, we sought to characterize neutrophil function in vivo using a lipopolysaccharide (LPS)-induced peritonitis model. In order to target neutrophils, we harvested peritoneal cells 24 h after LPS injection and examined neutrophil populations via flow cytometry. In response to LPS, the percent of GR1 + /CD11b + neutrophils in the lavage fluid was significantly higher in *Nf1*+/– mice when compared with WT mice (Fig. 1D). Similarly, the absolute number of GR1 + /CD11b + neutrophils per mL of lavage fluid was much higher in *Nf1*+/– mice confirming our in vitro observation that neurofibromin-deficient neutrophils exhibit a pro-survival and/or enhanced mobilization (Fig. 1E).

**Figure 2:**
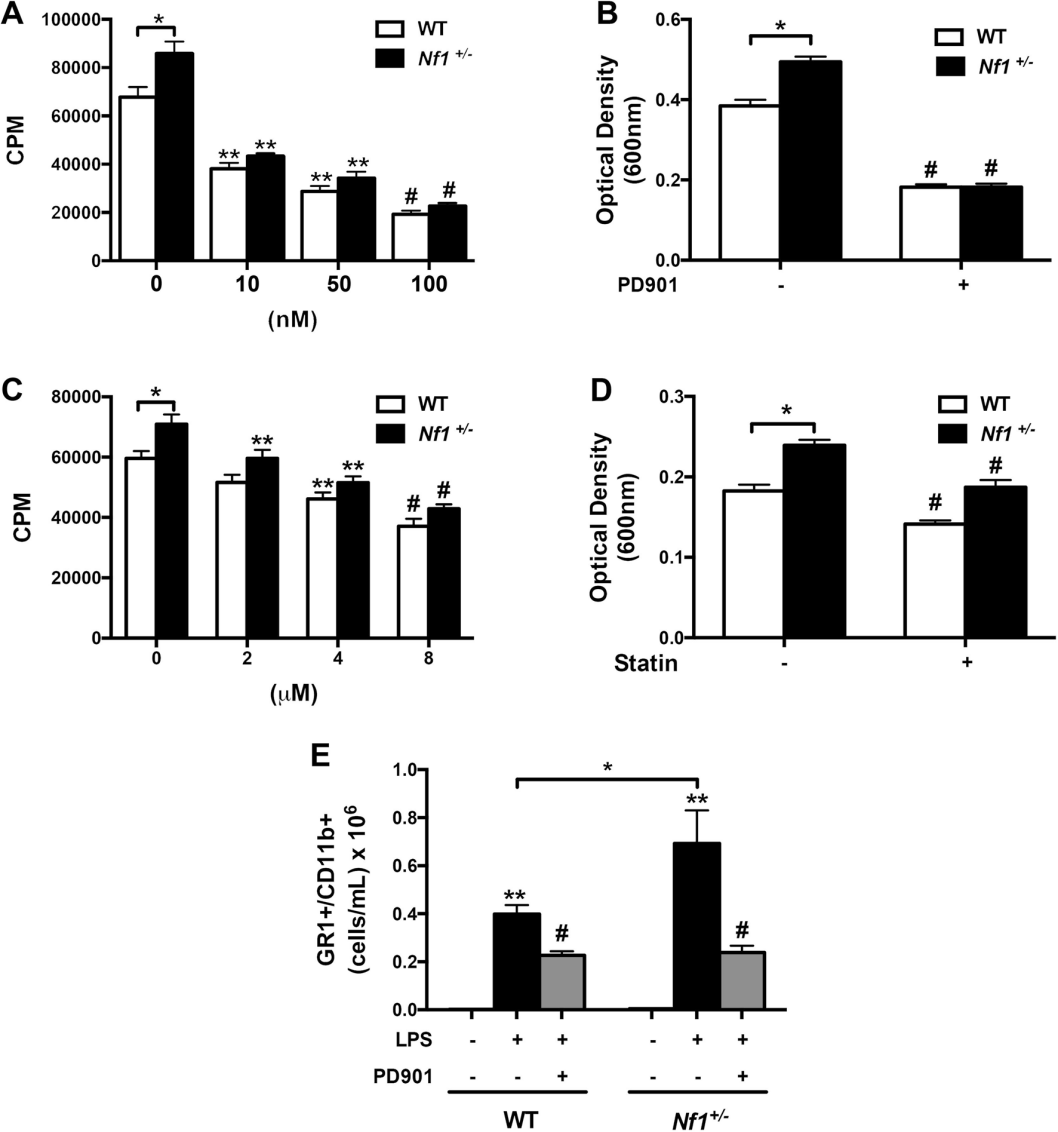
MEK inhibition impairs neurofibromin-deficient neutrophil function. (**A–D**). Quantification of WT (white bars) and *Nf1*+/– (black bars) neutrophil proliferation (**A **and** C**) and adhesion (**B **and** D**) in response to G-CSF in the presence of PD901 (**A **and** B**) or rosuvastatin (**C **and** D**). (**A**) Data represent thymidine incorporation reported as mean counts per minute (cpm) ± SEM, n = 4–5. *P < 0.01 for WT versus *Nf1*+/– neutrophils. **P < 0.001 for WT and *Nf1*+/– neutrophils versus WT and *Nf1*+/– neutrophils incubated with indicated concentration of PD901. #*P* < 0.01 for WT and *Nf1*+/– neutrophils incubated with 10 nM and 50 nM PD901 versus WT and *Nf1*+/– neutrophils incubated with 100 nM PD901. (**B**) Data represent mean optical density (600 nm) ± SEM, n = 4–5. *P < 0.001 for WT versus *Nf1*+/– neutrophils. #*P* < 0.001 for WT and *Nf1*+/– neutrophils versus WT and *Nf1*+/– neutrophils incubated with 50 nM PD901. (**C**) Data represent thymidine incorporation (cpm) ± SEM, n = 4–5. *P < 0.01 for WT versus *Nf1*+/– neutrophils. **P < 0.01 for WT and *Nf1*+/– neutrophils versus WT and *Nf1*+/– neutrophils incubated with indicated concentration of rosuvastatin. #*P* < 0.01 for WT and *Nf1*+/– neutrophils incubated with 2 μM rosuvastatin versus WT and *Nf1*+/– neutrophils incubated with 8 μM rosuvastatin. (**D**) Data represent mean optical density ± SEM, n = 4–5. *P < 0.001 for WT versus *Nf1*+/– neutrophils. #*P* < 0.01 for WT and *Nf1*+/– neutrophils versus WT and *Nf1*+/– neutrophils incubated with 10 μM rosuvastatin. (**E**) Quantification of absolute number of GR1 + /CD11b + neutrophils in peritoneal lavage fluid 48 h after LPS injection with/without PD901. *P < 0.001 for WT versus *Nf1*+/– mice. **P < 0.001 for WT and *Nf1*+/– mice without LPS injection versus WT and *Nf1*+/– mice provided LPS. #*P* < 0.001 for WT and *Nf1*+/– mice provided LPS versus WT and *Nf1*+/– mice provide LPS with daily PD901. Analysis by 2-way ANOVA.

Next, we sought to interrogate whether the enhanced function of neurofibromin-deficient neutrophils is mediated through the neurofibromin-MEK-ERK pathway. Cultured neutrophils from WT and *Nf1*+/– mice were incubated with a specific MEK inhibitor PD0325901 (PD901). We identified a dose-dependent reduction in *Nf1*+/– neutrophil proliferation and adhesion in response to PD901 and a similar but less robust response in WT neutrophils (Fig. 2A and B). Farnesylation is required for Ras activation and statins suppress farnesylation and other prenylation pathways^11^. Therefore, we incubated WT and *Nf1*+/– neutrophils with rosuvastatin and identified a similar dose-dependent reduction in *Nf1*+/– neutrophil proliferation and adhesion (Fig. 2C and D). Finally, we examined whether MEK inhibition impairs neutrophil function in vivo. To ensure adequate concentrations of PD901 in the peritoneum, we isolated peritoneal cells via lavage and probed for phosphorylated-ERK via immunoblot. Similar to our in vitro observations, PD901 exhibited a robust suppression of *Nf1*+/– neutrophil mobilization into the peritoneum with a similar but less robust response observed in WT neutrophils (Fig. 2E). Taken together, these data suggest that neurofibromin-Ras-MEK signaling is critical for neutrophil function, and both physiologic and constitutive Ras activation in neutrophils is sensitive to MEK inhibition.

### Early neutrophil depletion increases neointima size and suppresses circulating Ly6C^low^ monocyte frequency.

**Figure 3:**
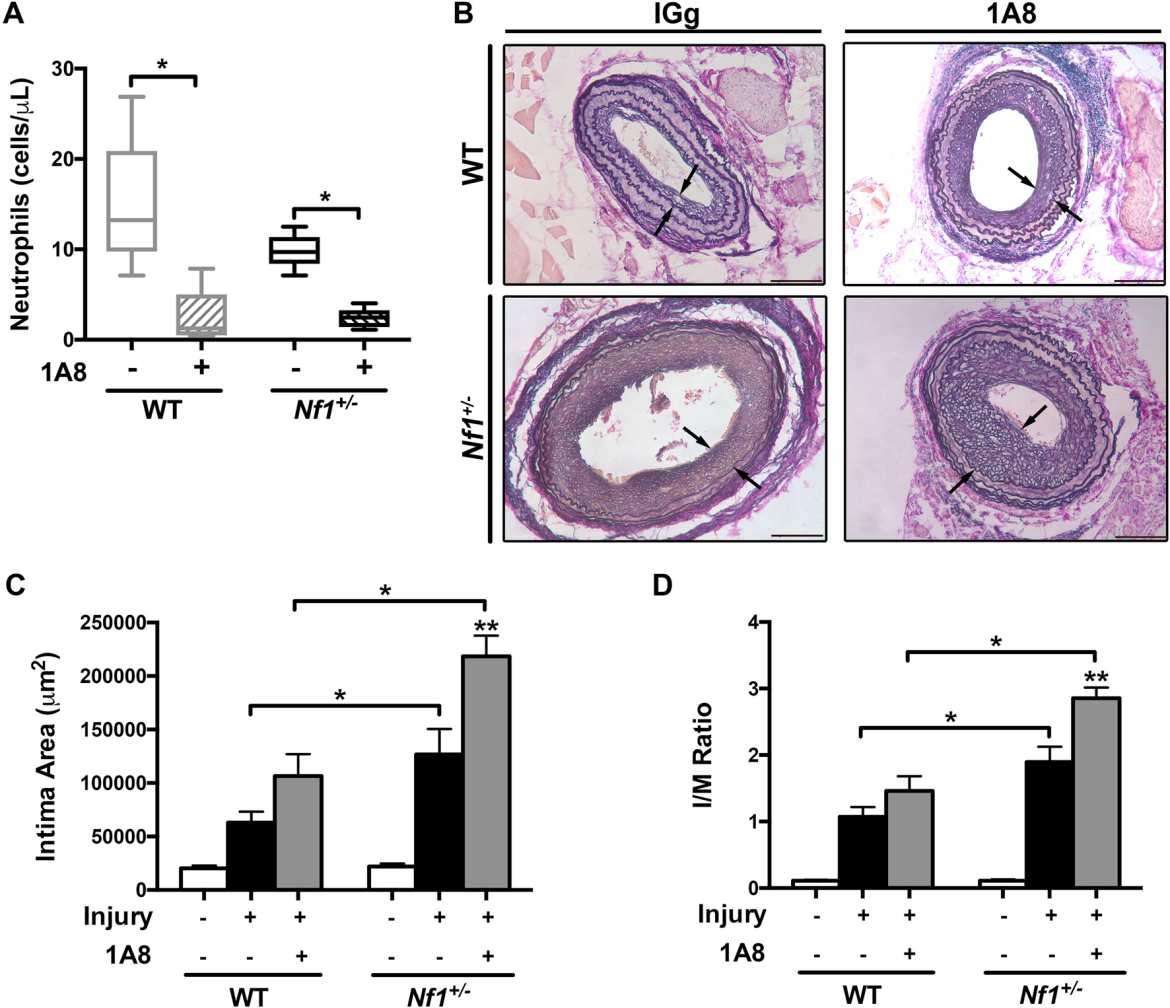
Neutrophil depletion increases neointima size in WT and *Nf1*+/– mice**. **(**A**) quantification of neutrophils after administration of 1A8 to WT (gray) and *Nf1*+/– (black) mice. *P < 0.01 for each comparison. (**B**)**.** Van Gieson-stained images of injured carotid arteries from WT and *Nf1*+/– mice provided IgG or 1A8. Black arrows indicate neointima boundary. Scale bars: 100 μm. (**C** and **D**) Data represent mean intima area (μm^2^) (**C**) and I/M ratio (**D**) ± SEM for uninjured and injured arteries from WT and *Nf1*+/– mice provided IgG or 1A8. n = 8–11 per group. *P < 0.01 for each comparison. **P < 0.01 for *Nf1*+/– mice receiving IgG versus *Nf1*+/– mice receiving monoclonal Ly6G antibody. Analysis by 2-way ANOVA.

Loss of neurofibromin enhances neutrophil and macrophage function and induces robust neointima formation, but the individual contributions of these differentiated myeloid cells to arterial stenosis in NF1 is poorly understood. In order to interrogate the role of neurofibromin-deficient neutrophils in neointima formation, we provided WT and *Nf1*+/– mice with a purified monoclonal antibody directed against the Ly6G antigen (1A8) once daily for the first 5 days after carotid artery injury. Importantly, Ly6G is considered to be a specific antigen differentiating neutrophils from monocytes^12^. As seen in Fig. 3A, WT and *Nf1*+/– mice provided 1A8 had significantly lower numbers of circulating neutrophils in the peripheral blood. Surprisingly, administration of 1A8 for 5 days after arterial injury resulted in much larger neointimas in both genotypes when compared with WT and *Nf1*+/– mice that received class-specific IgG (Fig. 3B–D). However, *Nf1*+/– neointimas were consistently larger than WT neointimas in both conditions.

**Figure 4:**
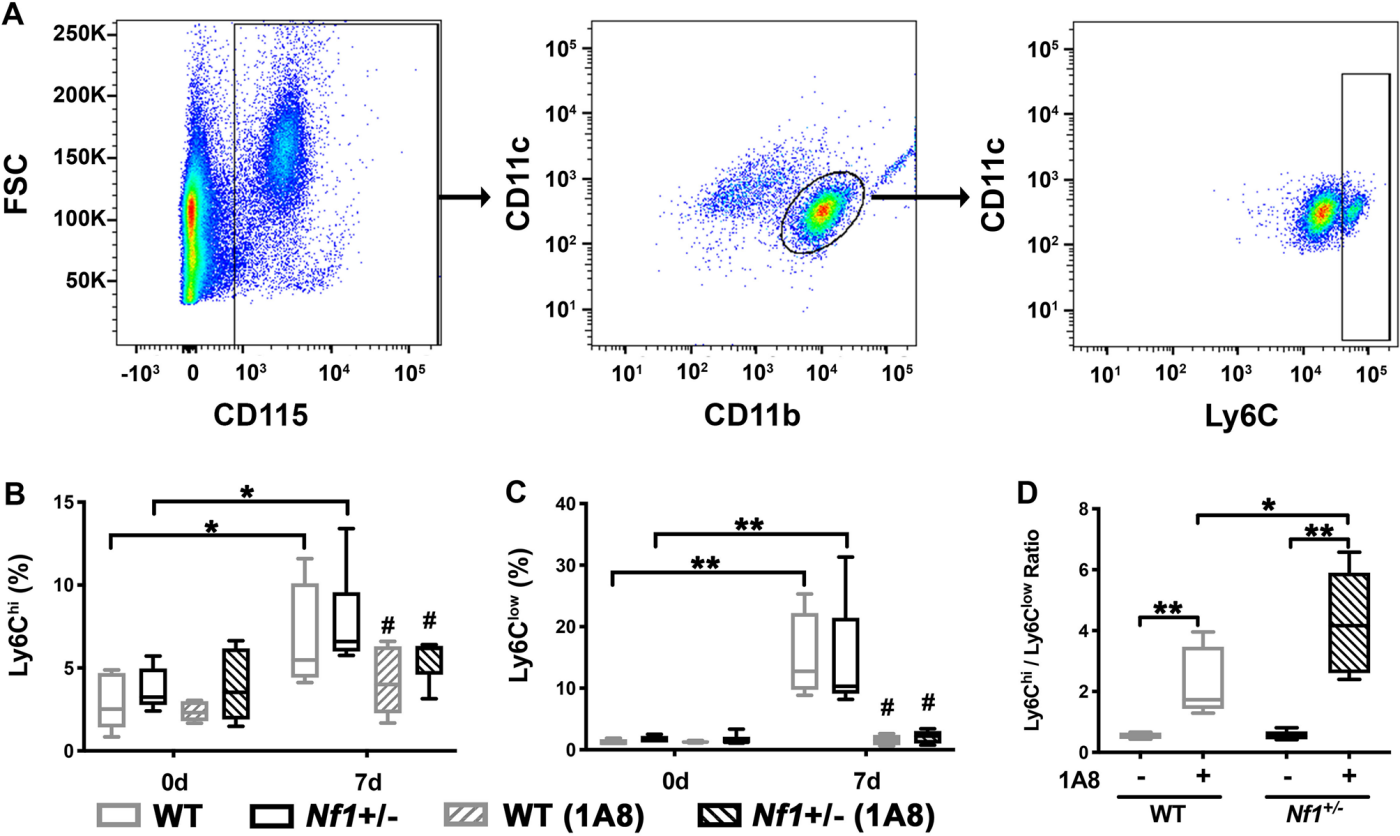
Early neutrophil depletion suppresses circulating Ly6C^low^ monocyte frequency. (**A**) Representative gating strategy to identify Ly6C^hi^ and Ly6C^low^ monocytes. (**B–D**) Quantitation of circulating Ly6C^hi^ (**B**), Ly6C^low^ (**C**) and Ly6C^hi^/Ly6C^low^ ratio (**D**) in WT (gray) and *Nf1*+/– (black) mice provided non-specific IgG or monoclonal Ly6G antibody. Boxes represent the 25th and 75th percentile with median value designated by horizontal line within each box. Whiskers represent the minimum and maximum values. *P < 0.05 and **P < 0.001 for each comparison. #*P* < 0.01 for WT and *Nf1*+/– mice provided non-specific IgG versus WT and *Nf1*+/– mice provided monoclonal Ly6G antibody. Analysis by 2-way ANOVA.

Neutrophils are recognized to be readily mobilized to sites of vascular injury and recruit other myeloid populations to sustain vascular repair and remodeling. A substantial body of evidence points to the role of specific murine monocyte subpopulations, denoted by the expression of Ly6C, to arterial remodeling and their presence in circulating blood is temporally-predictable following specific injury mechanisms^13–19^. Based on the larger neointimas observed after neutrophil depletion, we examined peripheral blood samples from all treatment cohorts to identify potential perturbations in Ly6C-expressing monocyte presence following arterial injury. As demonstrated by other groups, inflammatory Ly6C^hi^ monocytes are mobilized in the early phase of arterial inflammation in WT mice and a similar response was observed in *Nf1*+/– mice(Fig. 4A and B)^20^. However, the rise in Ly6C^hi^ monocytes in the days after arterial injury observed in control WT and *Nf1*+/– mice was blunted in WT and *Nf1*+/– mice provided 1A8 (Fig. 4B). More strikingly, patrolling Ly6C^low^ monocytes were mobilized following arterial injury in placebo-treated WT and *Nf1*+/– , but the frequency of Ly6C^low^ monocytes in peripheral blood was completely suppressed in both genotypes that received 1A8 (Fig. 4C). To further illustrate this relationship, the ratio of Ly6C^hi^ to Ly6C^low^ was significantly higher in WT and *Nf1*+/– mice provided 1A8 when compared with placebo treated WT and *Nf1*+/– mice, and this difference was exaggerated in 1A8-treated *Nf1*+/– mice (Fig. 4D). Together, this data suggests that neutrophils suppress early arterial remodeling and are critical to recruiting and/or maintaining Ly6C^low^ patrolling monocytes in the peripheral blood following arterial injury.

### Loss of neurofibromin in macrophages induce neointima formation via CCR2

**Figure 5:**
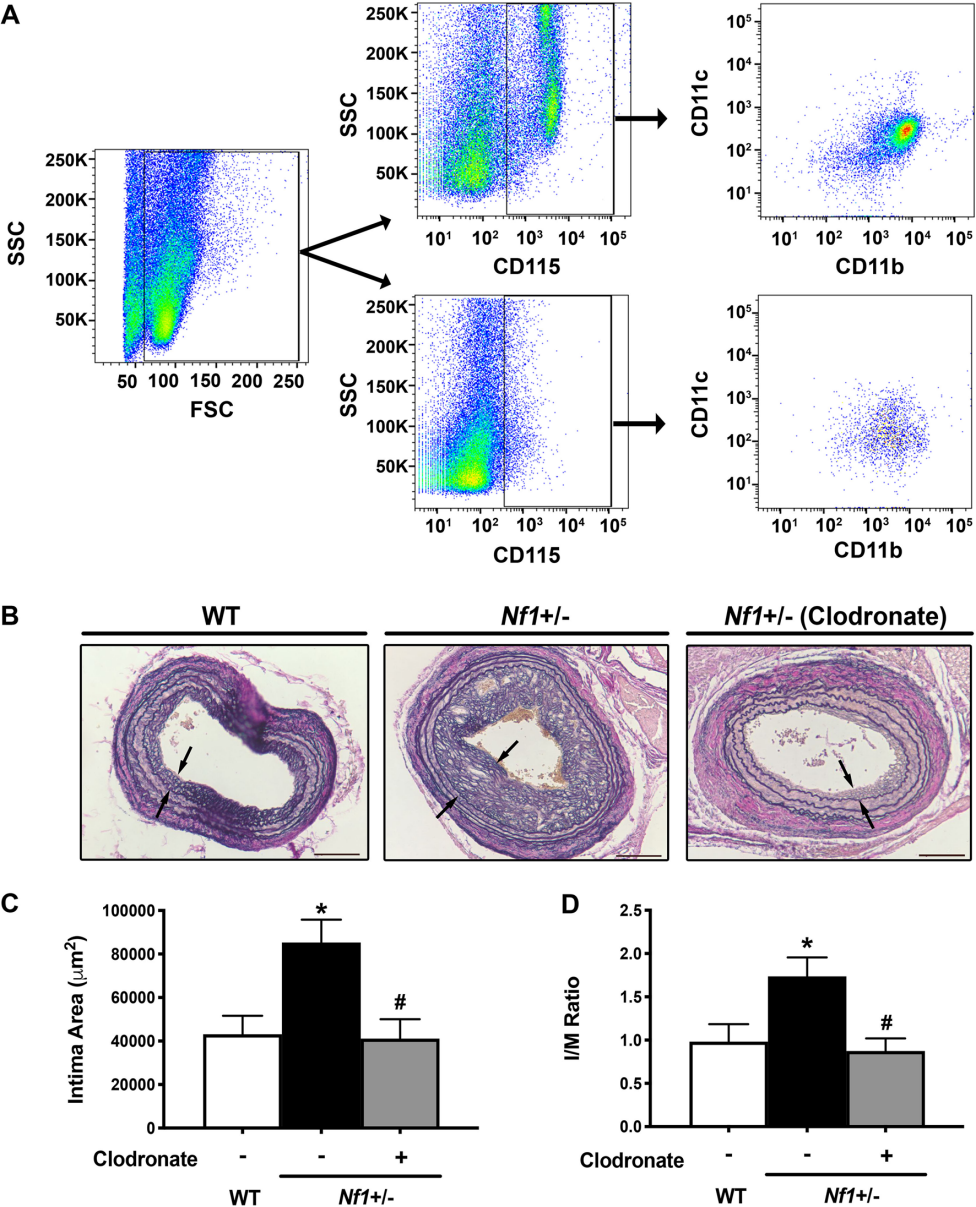
Monocyte/macrophage depletion decreases neointima size in *Nf1*+/– mice. (**A**) Representative gating strategy demonstrating depletion of CD115+ /CD11+ /CD11b+ monocytes. (**B**) Van Gieson-stained images of injured carotid arteries from WT and *Nf1*+/– mice provided liposomal clodronate or empty liposomes. Black arrows indicate neointima boundary. Scale bars: 100 μm. (**C **and** D**). Data represent mean intima area (μm^2^) (**C**) and I/M ratio (**D**) ± SEM, n = 8–11 per group. *P < 0.05 for WT versus *Nf1*+/– mice. #*P* < 0.05 for *Nf1*+/– mice receiving clodronate versus *Nf1*+/– mice receiving empty liposomes. Analysis by 1-way ANOVA.

Based on our observations that antibody-mediated neutrophil depletion enhanced neointima formation and altered circulating monocyte frequency in both WT and *Nf1*+/– mice, we suspected that neurofibromin-MEK-ERK activation in monocytes/macrophages is pathologically-linked to neointima formation. In order to specifically target monocytes/macrophages in the natural pathogenesis of arterial stenosis, we performed carotid artery ligation in WT and *Nf1*+/– mice and provided liposomal clodronate or control liposomes beginning one week after ligation (Fig. 5A). This treatment regimen corresponds with the delayed mobilization of monocytes in the peripheral blood and appearance of macrophages in the arterial wall. Arteries were harvested 14 days after injury (7 days of treatment) for analysis. While *Nf1*+/– mice provided empty liposome continued to display a robust neointima when compared with control WT mice, liposomal clodronate beginning 7 days after arterial injury strongly suppressed neointima formation in *Nf1*+/– mice (Fig. 5B–D).

**Figure 6:**
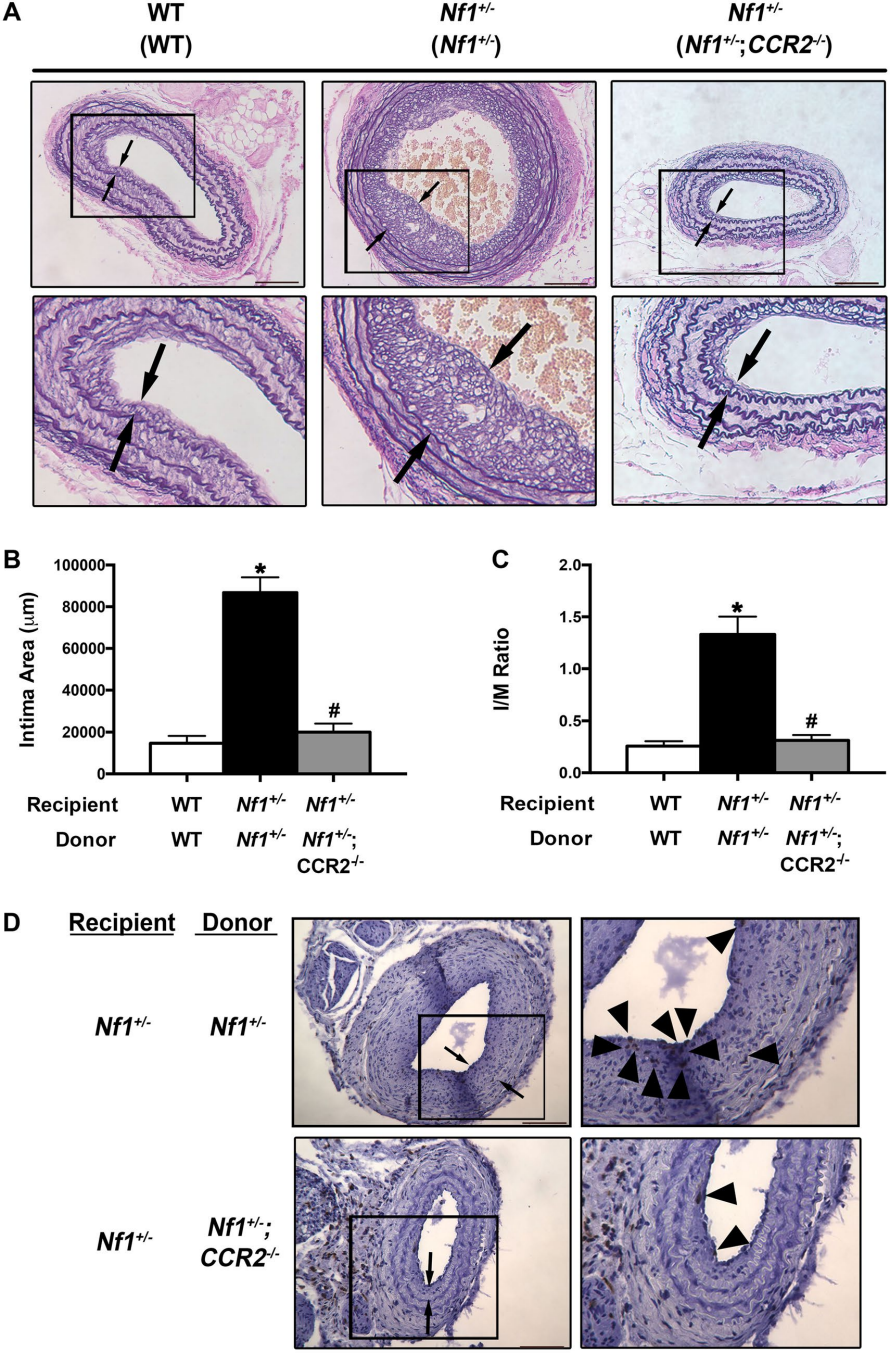
Monocytes/Macrophages drive neointima formation via CCR2. (**A**) Van Gieson-stained images of injured carotid arteries from WT reconstituted with WT bone marrow and *Nf1*+/– mice reconstituted with either *Nf1*+/– or *Nf1*+/– ;*CCR2*−/− bone marrow. Black arrows indicate neointima boundary. Black boxes identify area of injured artery that is magnified below. Scale bars: 100 μm. (**B** and **C**) Data represent mean intima area (μm^2^) (**B**) and I/M ratio (**C**) ± SEM, n = 9–10 per group. *P < 0.05 for WT mice reconstituted with WT bone marrow versus *Nf1*+/– mice reconstituted with *Nf1*+/– bone marrow. #*P* < 0.05 for *Nf1*+/– mice reconstituted with *Nf1*+/– bone marrow versus *Nf1*+/– mice reconstituted with *Nf1*+/– ;*CCR2*−/− bone marrow. Analysis by 1-way ANOVA. (**D**)**.** Representative images of Mac-3 staining in injured carotid arteries from *Nf1*+/– mice reconstituted with either *Nf1*+/– or *Nf1*+/– ;*CCR2*−/− bone marrow. Black arrows indicate neointima boundary. Black boxes identify area of injured artery that is magnified to the right. Scale bars: 100 μm.

Next, we sought to confirm these findings by employing techniques to impair monocyte/macrophage mobilization from the bone marrow rather than deplete circulating and resident cells via liposomal clodronate. Arising from the same progenitor population, granulocytes and monocyte/macrophages share many common markers and functions. However, C–C chemokine receptor type 2 (CCR2), the primary receptor for monocyte chemotactic protein-1 (MCP-1), is uniquely expressed on monocytes, but not granulocytes^21–23^. Therefore, we intercrossed *Nf1*+/– and *CCR2*−/− mice and transferred bone marrow cells isolated from *Nf1*+/– ;*CCR2*−/− mice into irradiated *Nf1*+/– mice, which were subjected to carotid artery injury. In response to arterial injury, *Nf1*+/– mice reconstituted with *Nf1*+/– bone marrow developed a robust neointima; however, deletion of *CCR2* from the bone marrow completely suppressed neointima formation in *Nf1*+/– mice (Fig. 6A–C). Further, neointima macrophage content was reduced in *Nf1*+/– mice reconstituted with *Nf1*+/– *;CCR2−/−* bone marrow (Fig. 6D). Thus, monocytes/macrophages appear to be principal drivers of neointima formation via a neurofibromin-MEK-ERK mediated pathway.

### Mature, but not early *Nf1*+/– neointima formation is sensitive to MEK inhibition.

**Figure 7:**
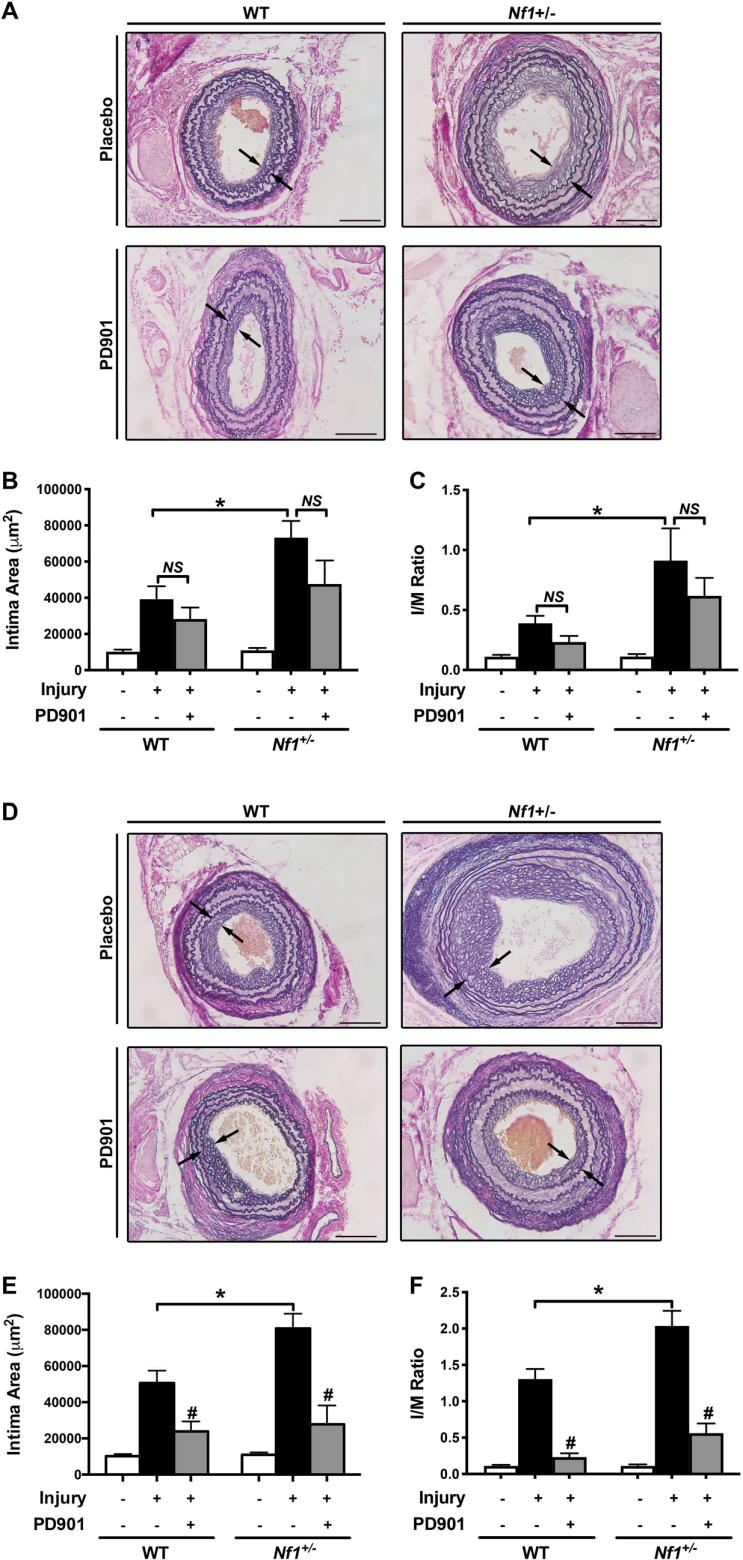
Mature, but not early neointima formation is sensitive to MEK inhibition (**A–F**) Representative photomicrographs (**A **and** D**) and quantification of intima area (**B **and **E**) and I/M ratio (**C **and **F**) of injured carotid arteries from WT and *Nf1*+/– mice treated with either placebo or PD901 for 7 days (**A–C**) or 14 days (**D–F**) Data represent the mean intima area (**B **and **E**) or I/M ratio (**C** and **F**) of 3 arterial cross Sects. (400, 800, and 1200 μm proximal to the ligation) ± SEM, *n* = 8–10 per group. *P < 0.05 for WT injured with placebo treatment versus *Nf1*+/– injured with placebo treatment. **#P** < 0.01 for injured with placebo treatment versus WT and *Nf1*+/– injured with 5 mg/kg/day PD901 treatment. Analysis by 2-way ANOVA.

Neutrophils and macrophages exhibit a temporal relationship in models of arterial remodeling wherein neutrophils appear in the hours and days after arterial injury and provide cues to monocytes/macrophages to localize to the site of vascular injury. This relationship may explain our previous observation that delaying initiation of the MEK inhibitor PD901 until 7 days after injury had a more profound inhibitory effect on neointima size when compared with sustained PD901 treatment beginning immediately after arterial injury^7^. In order to interrogate this possibility, we provided low-dose PD901 (5 mg/kg) to littermate WT and *Nf1*+/– mice immediately after carotid artery ligation and harvested arteries 7 days after injury to examine early neointima formation. Similar to murine arteries harvested 28 days after injury, *Nf1*+/– carotid arteries demonstrated a larger neointima compared to WT mice by 7 days after injury (Fig. 7A–C). Unlike our previous finding that sustained PD901 treatment inhibits neointima formation in WT and *Nf1*+/– mice 28 days after injury, administration of PD901 through the first 7 post-operative days resulted in a modest, but non-significant reduction in neointima size. To examine this nuanced relationship more closely, we extended our treatment regimen to 14 days in a separate cohort of WT and *Nf1*+/– mice. In contrast to a 7 day treatment regimen, 14 days of PD901 treatment beginning immediately after arterial injury significantly reduced neointima formation in both WT and *Nf1*+/– mice (Fig. 7D–F). Taken together, these data suggest that MEK signaling exert a temporal effect on VSMC proliferation and arterial stenosis and support our findings that neurofibromin-MEK-ERK activation in macrophages are pathogenically linked to neointima formation.

## Discussion

Neurofibromatosis type 1 is a heritable multisystem disease with a median age of death that is 15 years lower than the general population^24^. In addition to the cancer manifestations of NF1, cardiovascular disease is over-represented in this population and contributes to the early and excess mortality observed in persons with NF1^3^. NF1 vasculopathy is systemic, affecting both large and small vessels and commonly presents as renal and carotid artery stenosis, cerebral angiopathy, and arterial aneurysms. Emerging evidence suggests that persons with NF1 experience chronic inflammation and oxidative stress, which are closely tied to arterial disease in the general population and have been implicated in NF1 arteriopathy. Similar to persons with NF1, *Nf1*+/– mice develop robust arterial stenoses and aortic aneurysms and exhibit evidence of chronic inflammation and oxidative stress. Both persons with NF1 and *Nf1*+/– mice have increased circulating pro-inflammatory monocytes (CD16 + human and Ly6C^hi^ murine) and inflammatory cytokine concentrations (e.g. IL-6, IL-1β) in the absence of inflammatory stimuli or injury. Similar to findings in human monocytes isolated from persons with NF1, murine macrophages isolated from *Nf1*-mutant mice produce excessive quantities of superoxide, which has been identified in the arterial wall following ligation^6,10,25–28^. Thus, germline and lineage-restricted *Nf1* heterozygous mice represent a tractable platform for understanding NF1 vasculopathy.

Here, we define the role of neurofibromin in neutrophil function and uncover a surprising role for neutrophils in regulating NF1 arteriopathy. As is the case for other myeloid cell populations, loss of neurofibromin enhances neutrophil proliferation, migration, and adhesion through canonical MEK-ERK signaling^5,7^. PD901, a specific MEK1/2 inhibitor, impaired *Nf1*+/– neutrophil function in vitro and in vivo as did the Ras farnesylation inhibitor, rosuvastatin, demonstrating that suppression of Ras-MEK-ERK signaling impairs *Nf1*+/– neutrophil function. Since we previously showed that PD901 was efficacious in preventing neointima formation in *Nf1*+/– mice^7^ and suppresses *Nf1*+/– neutrophil function in vivo, we hypothesized that selective depletion of neutrophils would impair the early immune response and subsequent influx of macrophages during arterial stenosis leading to smaller neointimas in *Nf1*+/– mice. Surprisingly, we identified a much more robust neointima in both WT and *Nf1*+/– treated with 1A8 during the first week after carotid artery ligation. Previous published work shed light on the relationship between neutrophils and arterial stenosis. Administration of G-CSF to C57Bl/6 J mice four days prior to arterial injury reduced neointima formation in a wire-injury model although delaying treatment initiation until the time of endothelial denudation tempered the inhibitory effect of G-CSF on neointima size^29^. In a similar model, non-specific neutrophil depletion during the first week after arterial injury enhanced neointima size and reduced macrophage content within the arterial wall^30^. Further, neutropenic mice lacking neutrophil-derived serine proteases or reduced granulocyte content did not display larger neointimas; rather, deletion of cathelicidin, a monocyte chemoattractant secreted by neutrophils, in the bone marrow resulted in larger neointimas reminiscent of those identified in mice with early neutrophil depletion^30^. Interestingly, neutropenic mice displayed reduced adhesion of Ly6C + monocytes to blood vessels and injection of cathelicidin restored Ly6C + monocyte adhesion^31^. The authors conclude that neutrophils enable monocyte homing during vascular repair.

Under physiologic conditions, Ly6C expressing monocytes are mobilized into the circulation within the first week after arterial injury^32^. Both WT and *Nf1*+/– mice displayed a similar two-fold increase in Ly6C^hi^ monocytes and a 15-fold increase in Ly6C^low^ monocytes within the first 7 days after injury; however, neutrophil depletion significantly blunted the early phase of Ly6C + monocyte recruitment. While Ly6C^hi^ monocyte mobilization was modestly lower in both neutrophil-depleted WT and *Nf1*+/– mice, the frequency of Ly6C^low^ monocytes in both genotypes following 1A8 treatment was completely blunted and nearly identical to pre-injury levels. Monocyte CCR2 co-expresses with Ly6C and an increasing body of evidence suggests that Ly6C^hi^;CCR2 + monocytes are initial responders to inflammatory cues and give rise to Ly6C^low^ monocytes, which generally express low levels of CCR2^33,34^. The significant reduction in intravascular Ly6C^low^ monocytes following neutrophil depletion may be explained by a lack of secreted neutrophil-derived chemoattractants (eg cathepsins and MCP-1) and delays in the second wave of the immune response characterized by increased circulating monocyte frequency^35,36^. During early inflammation states, neutrophils release soluble interleukin-6 (IL-6) receptor which binds IL-6 to trigger the release of MCP-1 from neighboring EC. In turn, MCP-1 initiates the recruitment of Ly6C^hi^ monocytes via CCR2^37,38^. Neutrophil depletion slows the release of MCP-1 from EC and subsequent Ly6C^hi^ recruitment and ultimately the generation of circulating Ly6C^low^ monocytes. This line of thinking is intriguing since several independent groups have shown that circulating IL-6 concentrations are increased in persons with NF1 when compared with age- and sex-matched controls^6,26^. High circulating IL-6 concentrations may enhance monocyte egress from the bone marrow and other niches, which supports the monocytosis observed in persons with NF1 and the increased numbers of Ly6C^hi^ monocytes in *Nf1*-mutant mice^5,6,10^.

While this hypothesis is certainly plausible since intravascular Ly6C^hi^ monocyte number is reduced following neutrophil depletion, it must be noted that neutrophils are known to specifically interact with Ly6C^low^ monocytes and assist in retaining these monocytes within the vascular space^39,40^. Mice deficient in CX3CR1, a marker of Ly6C^low^ monocytes responsible for patrolling behaviors, have low circulating Ly6G + neutrophils^41^. During acute inflammation, interaction time between intravascular neutrophils and Ly6C^low^ monocytes increases; however, pharmacologic or genetic disruption of CX3CR1 signaling impairs this interaction and cues Ly6C^low^ monocytes to produce inflammatory cytokines including TNF-α^42^. The absence of intravascular neutrophils allows Ly6C^low^ monocytes to leave the vascular space and may encourage remaining Ly6C^low^ monocytes to produce inflammatory cytokines to exacerbate inflammation. Conversely, Ly6C^low^ monocytes secrete growth factors that enhance neutrophil migration suggesting that neutrophil-monocyte interactions are necessary to limit excessive inflammation^41^. The absence of intravascular Ly6C^low^ monocytes is critical to interpreting the enhanced neointima in neutrophil-depleted mice since Ly6C^low^ monocytes display several anti-inflammatory and reparative features and are largely seen as protective against vascular inflammation^15,19^. Clearly, the interaction between blood neutrophils and Ly6C^low^ monocytes is reciprocal and disruption of these relationships and feedback loops creates an environment for unrestricted inflammation.

Based on the evidence that selective neutrophil depletion enhances neointima size and reduces circulating Ly6C-expressing monocytes, we utilized liposomal clodronate to specifically deplete macrophages after the acute phase of arterial injury. Macrophage depletion beginning 7 days after arterial injury demonstrated similar efficacy against neointima formation as delayed or sustained PD901 treatment. These data suggest that neurofibromin deficiency and subsequent MEK-ERK activation in monocytes/macrophages are instrumental in promoting arterial remodeling and neointima formation. Our findings are congruent with previous reports demonstrating that administration of macrophage colony stimulating factor (M-CSF) after arterial injury increases neointima size and macrophage content in the injured vessel^43^. Conversely, modulation of macrophage-generated ROS or cytokines attenuates neointima formation^44,45^. To confirm these findings, we transferred *Nf1*+/– ;*CCR2*−/− bone marrow into *Nf1*+/– mice and subjected them to arterial injury. Importantly, CCR2 is expressed on monocytes and macrophages, but not neutrophils^46,47^. Similar to previous observations, transfer of *Nf1*+/– into *Nf1*+/– mice resulted in a large neointima and loss of *CCR2* in bone marrow cells completely suppressed neointima formation in *Nf1*+/– mice. Together with our neutrophil and macrophage depletion data, monocytes/macrophages appear to drive VSMC proliferation and neointima formation in *Nf1*+/– mice. Both CCR2 and its cognate antigen CCL2/MCP-1 are linked to human arterial stenosis and genetic and pharmacologic disruption of MCP-1/CCR2 signaling effectively suppresses arterial remodeling in animal models^48,49^. Unfortunately, pharmacologic targeting of either MCP-1 or CCR2 in human trials have failed to demonstrate efficacy with this approach and likely reflects complex interactions between monocytes, vascular wall cells (e.g. EC and VSMC) as well as other hematopoietic cells including neutrophils.

Finally, we sought to reconcile the protective role that neutrophils appear to play in limiting neointima size in *Nf1*-mutant mice with our observations that enhanced MEK-ERK signaling enhances *Nf1*+/– neutrophils function. On the one hand, MEK activation and the resultant pro-survival phenotype observed in neurofibromin-deficient neutrophils should limit neointima size in *Nf1*+/– mice and pharmacologic inhibition of MEK with PD901 would hypothetically impair neutrophil function leading to larger neointimas. However, our previous finding that delaying initiation of PD901 until one week after arterial injury is more effective than initiating therapy at the time of ligation suggests that MEK-ERK inhibition exerts a nuanced and temporal effect on myeloid cell populations linked to arterial remodeling. In order to more fully understand the temporal relationship between PD901 initiation and neointima formation, we provided WT and *Nf1*+/– mice with low dose PD901 for 7 or 14 days following arterial injury and harvested carotid arteries for analysis. Short duration PD901 treatment resulted in a modest, but nonsignificant reduction in neointima size in *Nf1*+/– mice. Increasing the duration of treatment to 14 days completely suppressed neointima formation in both WT and *Nf1*+/– mice. While direct comparisons were not part of the pre-planned analysis of this experiment, it must be noted that *Nf1*+/– neointimas continue to expand between 7 and 14 days after injury while WT neointimas experience less growth during this same time period. It is intriguing to hypothesize that neurofibromin-deficient monocytes recruited during this window give rise to infiltrating macrophages that drive the pathogenesis of NF1 arteriopathy and therefore targeted MEK inhibition during this window is more effective than early treatment. Taken together, these observations suggest a therapeutic window for MEK activation exists after the early phase of arterial remodeling following arterial injury.

Here, we provide the first evidence that neutrophils carrying inactivating mutations in *Nf1* display a pro-survival phenotype mediated through Ras-MEK signaling. Further, our data suggests that MEK signaling in neutrophils is critical to maintain regulatory monocytes within the vascular space during arterial remodeling and that neurofibromin-MEK activation in neutrophils may play a protective role to counteract the excessive remodeling driven by inflammatory monocytes and macrophages. While these observations are intriguing, they also raise a cautionary sign of the complexity of myeloid cell interactions and point to a need for cell-specific therapeutic approaches for persons with NF1.

## Materials and methods

### Animals

Protocols were approved by Institutional Animal Care and Use Committee at Augusta University. *Nf1*+/– mice were obtained from Tyler Jacks (Massachusetts Institute of Technology, Cambridge, MA) and backcrossed 13 generations into the C57BL/6 J strain^50^. Male WT and *Nf1*+/– mice between 12 and 15 weeks of age were used for experiments. *CCR2* knockout mice were purchased from The Jackson Laboratory (#4999) and maintained on C57BL/6 strain. *Nf1*+/– mice were intercrossed with *CCR2* knockout mice to produce *Nf1*+/– ;*CCR2*−/− mice. In some experiments, WT and *Nf1*+/– mice were lethally irradiated and reconstituted with bone marrow from WT, *Nf1*+/– , and *Nf1*+/– ;*CCR2*−/− mice. All methods were carried out in accordance with relevant guidelines and regulations by the American Veterinary Medical Association Guidelines for the Euthanasia of Animals. All studies are compliant with the ARRIVE guidelines for reporting experiments involving animals.

### Carotid artery ligation

Carotid artery injury was induced by ligation of the right common carotid artery as described^5^. Briefly, mice were anesthetized by inhalation of an isoflurane (2%)/oxygen (98%) mixture. Under a dissecting scope, the right carotid artery was exposed through a midline neck incision and ligated proximal to the bifurcation using a 6–0 silk suture. The contralateral carotid artery was sham ligated as a control. Mice were administered 15 μg of buprenorphine (IP) following the procedure and recovered for 28 days. In some experiments, *Nf1*+/– and WT mice were administered 5 mg/kg of PD0325901 (Selleck Chemicals, IC_50_: 0.33 nM) or vehicle via oral gavage. To deplete neutrophils, *Nf1*+/– and WT mice received a monoclonal Ly6G (1A8, Biolegend) antibody (0.5 mg) or IgG control via IP injection at the time of carotid artery ligation and at 24-h intervals for 5 total doses. To deplete monocyte/macrophages, liposomal clodronate or control liposomes (Liposoma) were injected via tail vein seven days after carotid artery ligation and once every 48 h for 4 total doses.

### Morphometric analysis

Van Gieson-stained arterial cross Sections 400, 800, and 1,200 μm proximal to the ligation were analyzed for neointima formation using Image J (NIH, Bethesda, MD). Lumen area, area inside the internal elastic lamina (IEL), and area inside the external elastic lamina (EEL) were measured for each cross section. To account for potential thrombus formation, arteries containing significant thrombus (> 50% lumen occlusion) at 400 μm proximal to the ligation were excluded from analysis. The number of excluded arteries was not different between experimental groups. Representative photomicrographs for each figure are taken from arterial cross sections between 600 and 1200 μm proximal to the bifurcation. Intima area was calculated by subtracting the lumen area from the IEL area, and the media area was calculated by subtracting the IEL area from the EEL area. Intima/Media (I/M) ratio was calculated as intima area divided by media area.

### Generation of chimeric animals

Bone marrow mononuclear cells (BMMC) were harvested from WT, *Nf1*+/– , and *Nf1*+/– ;*CCR2*−/− mice and 5 × 10^6^ BMMC were resuspended in 300μL of Dulbecco's Modified Eagle Medium (DMEM) for infusion. Next, *Nf1*+/– and WT mice were conditioned by gamma irradiation with a single dose of 9.5 Gy. Conditioned *Nf1*+/– mice were reconstituted with BMMC isolated from *Nf1*+/– ;*CCR2*−/− mice. Separately, conditioned *Nf1*+/– and WT mice were reconstituted with *Nf1*+/– and WT BMMC, respectively, as controls. BMMC were infused immediately after gamma irradiation via tail vein injection. The source of circulating hematopoietic cells in recipient mice was identified by use of CD45.1/45.2 congenic strains. Donor mice expressed the leukocyte antigen CD45.1 and recipient mice expressed CD45.2. After a 6-week recovery period, blood (100µL) was sampled from the tail vein and assessed for engraftment using anti-CD45.1-PE-Cy7 and anti-CD45.2-FITC (Fisher) on a BD LSRII flow cytometer with a 405-nm violet laser, a 488-nm blue laser, and a 633-nm red laser. Mice expressing > 80% CD45.1 positive leukocytes in the peripheral blood underwent carotid artery ligation as described.

### Hemavet and polychromatic flow cytometry

Blood samples were obtained via tail vein and collected into EDTA tubes (BD Biosciences, San Jose, CA) for flow cytometry or for white blood cell count using the Hemavet 950 (Drew Scientific, Dallas, TX). For flow cytometry, red cell lysis was performed and cells were washed in 2% fetal bovine serum (FBS). Samples were centrifuged, resuspended in 2% FBS, and incubated with murine Fc Blocking Reagent (Miltenyi Biotec, Cologne, Germany) on ice. Samples were then stained with CD45-APC (Biolegend), CD115-PE (Fisher), CD11b-APC-Cy7 (BD Biosciences), CD11c-PerCP-Cy5.5 (BD Biosciences), Ly6C-PE-Cy7 (BD Biosciences), Ly-6B.2-FITC (F7/4; Bio-Rad), TER119-Pac Blue (Fisher), and LIVE/DEAD violet fixable dead cell stain (Invitrogen, Grand Island, NY). Antibodies were tittered for optimal staining and “fluorescence-minus-one” (FMO) gating controls were performed to determine true positive and negative events. Stained samples were acquired on a BD LSRII flow cytometer equipped with a 405-nm violet laser, 488-nm blue laser, and 633-nm red laser. At least 100,000 events were collected for samples. Data were collected uncompensated and analyzed using FlowJo software version 8.7.3 (Tree Star).

### Isolation of bone marrow-derived neutrophils and characterization

Murine bone marrow–derived neutrophils were generated from 6- to 8-week-old WT and *Nf1*+/– femurs, tibias, and iliac crest. Briefly, bone marrow cells were flushed into 50-mL Falcon tubes using syringe needle and Iscove modified Dulbecco medium (IMDM; Invitrogen). Cells were collected by centrifugation at 800* g* for 5 min (Beckman Coulter) and RBCs were lysed with RBC lysis buffer containing 155 mM ammonium chloride, 10 mM potassium bicarbonate, and 0.1 mM ethylenediaminetetraacetic acid for 5 min. Low-density bone marrow (LDBM) cells were isolated by density gradient centrifugation using histopaque 1083 (Sigma-Aldrich). LDBM cells were cultured in IMDM, 10% fetal bovine serum supplemented with 1% penicillin/streptomycin, and in the presence of 1 ng/mL granulocyte colony-stimulating factor (G-CSF) and 5 ng/mL interleukin-3 (PeproTech).

Proliferation was assessed by incorporation of radioactive thymidine in WT and *Nf1*+/– BM-derived neutrophils. Briefly, WT and *Nf1*+/– neutrophils (5 × 10^4^ cells) were serum-starved for 12 h and placed in a 96-well plate in 200 μl starvation media in either the absence or presence of G-CSF (10 ng/mL). After 24 h, cells were pulsed with 1.0 μCi (0.037 MBq) tritiated thymidine for 6 h, harvested, and thymidine incorporation was determined as counts per minute (cpm). In some experiments, cells were incubated with PD325901 or rosuvastatin at indicated concentrations. All experiments were performed in triplicate.

For neutrophil adhesion, 1 × 10^5^ cells were added to each well of a flat-bottom 96-well polystyrene plate coated with fibronectin (20 μg/ml) and incubated at 37 °C for indicated length of time. Unbound cells were removed by aspiration and adherent cells were washed, fixed with 3.5% formaldehyde, and stained with 0.1% crystal violet. The stain was eluted with 10% acetic acid and absorbance was determined at 600 nm using a microplate reader (Spectramax 250; Molecular Devices). In some experiments, cells were co-incubated with PD325901 or rosuvastatin at indicated concentrations. All experiments were performed in triplicate.

For neutrophil migration, WT and *Nf1*+/– neutrophils (2.5 × 10^5^ cells) were placed in the upper chamber of the transwell (3 μM pore filter; Costar) and allowed to migrate toward the bottom of the transwell containing G-CSF (10 ng/mL). After 12 h, non-migrated cells in the upper chamber were removed with a cotton swab and migrated cells attached to the bottom surface of the membrane were stained with 0.1% crystal violet dissolved in 0.1 M borate, pH 9.0, and 2% ethanol for 5 min at room temperature. The number of migrated cells was determined in 5 random fields with an inverted microscope using a 20X objective lens. All experiments were performed in triplicate.

### Statistical analysis

All values are presented as mean or percent ± S.E.M. Cell proliferation and migration were analyzed by 2-way ANOVA with Tukey’s post-hoc test for multiple comparisons. All experiments were performed in triplicate. Intima area and I/M ratio analysis was assessed by 1-way and 2-way ANOVA with Tukey’s post-hoc test for multiple comparisons. Analysis was performed using GraphPad Prism version 5.0d. *P* < 0.05 were considered significant.

## Data Availability

All data generated or analyzed during this study are included in this published article.
